# Ion Tracks and Nanohillocks Created in Natural Zirconia Irradiated with Swift Heavy Ions

**DOI:** 10.3390/ma17030547

**Published:** 2024-01-23

**Authors:** Norito Ishikawa, Shoma Fukuda, Toru Nakajima, Hiroaki Ogawa, Yuki Fujimura, Tomitsugu Taguchi

**Affiliations:** 1 Nuclear Science and Engineering Center, Japan Atomic Energy Agency (JAEA), Tokai 319-1195, Ibaraki, Japan; ogawa.hiroakixx@jaea.go.jp (H.O.); fujimura.yuki@jaea.go.jp (Y.F.); 2 Tono Geoscience Center, Japan Atomic Energy Agency (JAEA), Toki 509-5102, Gifu, Japan; fukuda.shoma@jaea.go.jp (S.F.); nakajima.toru@jaea.go.jp (T.N.); 3 Foundational Quantum Technology Research Directorate, National Institutes for Quantum Science and Technology (QST), Takasaki 370-1292, Gunma, Japan; taguchi.tomitsugu@qst.go.jp

**Keywords:** swift heavy ion, hillocks, ion tracks, ion irradiation, TEM, 78.67.Bf, 61.80.Jh, 61.46.Df, 34.50.Dy, 79.20.Rf

## Abstract

Natural monoclinic zirconia (baddeleyite) was irradiated with 340 MeV Au ions, and the irradiation-induced nanostructures (i.e., ion tracks and nanohillocks) were observed using transmission electron microscopy. The diameter of the nanohillocks was approximately 10 nm, which was similar to the maximum molten region size calculated using the analytical thermal spike model. Ion tracks were imaged as strained regions that maintained their crystalline structure. The cross-sections of most of the ion tracks were imaged as rectangular contrasts as large as 10 nm. These results strongly indicated that the molten region was recrystallized anisotropically, reflecting the lattice structure. Furthermore, low-density track cores were formed in the center of the ion tracks. The formation of low-density track cores can be attributed to the ejection of molten matter toward the surface. A comparison of the ion tracks in the synthetic zirconia nanoparticles and those in larger natural zirconia samples showed that the interface between the strained track contrast and the matrix was less clear in the former than in the latter. These findings suggest that the recrystallization process was affected by the size of the irradiated samples.

## 1. Introduction

An understanding of the damage formation mechanisms in irradiated materials is essential in the field of nuclear material research. Because fission fragments have an initial energy in the order of 100 MeV, swift heavy ions (SHIs) in the MeV–GeV energy range supplied by ion accelerators can be used to investigate radiation damages due to nuclear fission events. When SHIs are irradiated onto ceramic materials, columnar defect regions called ion tracks are created along the ion paths [1,2,3,4]. SHIs lose energy mainly via electronic energy loss within a material. Therefore, the electronic stopping power is the most important parameter for describing the ion track formation process. Many studies have found that the threshold stopping power to form ion tracks is closely related to the melting temperature of the target materials. The thermal spike model, which assumes local melting near the ion trajectory, is typically used to interpret ion track formation [1,2,3].

Transmission electron microscopy (TEM) results of SHI-irradiated ceramics [5,6,7,8,9,10] have shown that, in many ceramics, ion tracks exhibit common features, such as (1) an amorphous structure, (2) a circular cross-section, and (3) a clear interface between the amorphized region and undamaged matrix. These track features strongly suggest that local melting (solid–liquid phase transition) and successive rapid cooling (quenching) cause the formation of amorphized ion tracks. Such amorphized ion tracks are formed in SHI-sensitive materials called amorphizable materials (e.g., Y_3_Fe_5_O_12_ [7,11], ZrSiO_4_ [8,12,13], and LiNbO_3_ [9,14,15]).

In contrast to the amorphous features of ion tracks in amorphizable materials, different features can be observed in the ion tracks in so-called nonamorphizable materials (e.g., Al_2_O_3_ and CeO_2_) [16,17,18,19,20,21]. The distinct features of ion tracks formed in nonamorphizable materials can be summarized as follows: (1) the ion tracks are not amorphized, and (2) their lattice structure is almost maintained, and point defects are introduced near the ion paths. These features can be reasonably interpreted by assuming partial recrystallization after local melting [19,20,21].

In many cases, nanometer-sized surface protrusions (nanohillocks) can also be formed by SHI irradiation [22,23,24,25,26,27,28,29,30,31]. Because a nanohillock is formed as a part of an ion track, it is also called a surface ion track. According to our previous studies [30,31], the diameter of nanohillocks appears to always be similar to the maximum diameter of the molten region, irrespective of the amorphizability of the materials. Therefore, it appears that the size of the nanohillocks reflects the size of the melt. In the present study, the validity of this hypothesis is strengthened in zirconia samples.

The vital importance of recrystallization has also been demonstrated by previous studies on molecular dynamics (MD) simulations of Al_2_O_3_ and MgO [19,20]. In particular, in MgO, almost no damaged region remains because of rapid recrystallization. In the present study, the morphology of ion tracks is investigated to clarify how the molten region recrystallizes in zirconia. Furthermore, the size of the nanohillocks is compared with that of the melt. This approach allows us to reveal the melting and successive recrystallization processes.

Among many nonamorphizable materials, monoclinic zirconia is an interesting material that exhibits intriguing features of ion tracks. In a previous study on natural zirconia [32], the rectangular cross-section of the ion tracks was observed using TEM. The rectangular shape of zirconia ion tracks appears to be a distinct feature, because ion tracks in many ceramics have a circular cross-section. The same research group also found that stabilized tetragonal track regions are induced in a monoclinic matrix of natural zirconia by a single SHI impact [33]. Phase transformation in monoclinic zirconia irradiated with SHIs has been extensively investigated [34,35,36,37,38,39,40]. Therefore, monoclinic zirconia can be an intriguing material for investigating the mechanism of SHI-induced melting and recrystallization processes. In this study, ion tracks and nanohillocks are further investigated in natural monoclinic zirconia using TEM. The effect of particle size on the morphology of irradiation-induced nanostructures is also examined by investigating the irradiation-induced nanostructures of zirconia nanoparticles.

## 2. Experiments

The main sample in this study is natural monoclinic zirconia (baddeleyite), which was collected from a placer deposit in South Africa. The sample was separated using standard mineral separation techniques with a magnetic separator and heavy liquid. Firstly, the samples were observed using an optical microscope, and it was confirmed that the materials of natural zirconia had size ranges between 100 and 200 μm. Secondly, the samples were finely ground using an agate mortar and pestle. Thirdly, it was confirmed using an X-ray diffractometry MXP model 1030 (MAC Science Co., Ltd., Yokohama, Japan) that the XRD peaks of the ground samples can be assigned by those of the monoclinic crystal structures. The XRD patterns of the natural zirconia after grinding will be presented later. These finely ground samples were dispersed in ethanol in an ultrasonic bath, and ethanol was then dropped on a 3 mm diameter 200-mesh copper grid covered with porous carbon films. The grid was air-dried at room temperature to prepare the TEM sample. 

TEM samples of nanoparticle zirconia were also prepared to examine the effect of particle size on the morphology of irradiation-induced nanostructures. Synthetic zirconia, ZrO_2_ (98%), in nanoparticle form was purchased from the Kojundo Chemical Laboratory Co. Ltd., Saitama, Japan. It was confirmed that the XRD peaks of the as-purchased zirconia nanoparticles can be assigned by those of the monoclinic crystal structures. The XRD patterns of the as-purchased zirconia nanoparticles will be presented later. The as-purchased samples were dispersed in ethanol in an ultrasonic bath, and ethanol was dropped on a 3 mm diameter 200-mesh copper grid covered with porous carbon films. The grid was air-dried at room temperature. The size of the zirconia nanoparticles was mostly in the range of 30–80 nm.

The samples on the TEM grids were irradiated with 340 MeV Au ions at room temperature in a tandem accelerator at the JAEA-Tokai (Japan Atomic Energy Agency, Tokai Research and Development Center, Tokai, Japan). According to the SRIM-2010 code [41,42], the S_e_ of a 340 MeV Au ion beam in zirconia was estimated to be 36.1 keV/nm. We chose the irradiation condition so that ion tracks were formed in both the natural zirconia and zirconia nanoparticles. In the present study, the ion (340 MeV Au) was chosen so that ion tracks could be definitely formed by using 340 MeV Au, which has a much higher electronic stopping power (S_e_ = 36.1 keV/nm) than the ions used in the previous studies.

Irradiation was performed under two different incidence angles: the normal direction and 45° relative to the normal direction. Irradiation in the normal direction was performed to observe the cross-section of the ion tracks, whereas that at an inclined incidence was performed to observe the line-like contrasts of the ion tracks and side views of the nanohillocks. The ion fluence was in the range of 1 × 10^11^–3 × 10^11^ ions/cm^2^. The ion fluence was chosen so that independent track contrasts could be imaged. If the ion fluence is higher than this range, there is a higher chance of the overlapping of ion tracks. If the ion fluence is lower than this range, it is difficult to image multiple independent ion tracks in one grain. The irradiation flux was in the range of 1 × 10^8^–2 × 10^8^ ions/cm^2^/s. TEM images were captured using a 2100F (JEOL Ltd., Tokyo, Japan) operated at 200 kV. In order to analyze the crystal orientation, the VESTA software Version 3.5.8 was used for visualizing the crystallographic structure of the monoclinic zirconia [43].

## 3. Results

### 3.1. XRD Patterns of Unirradiated Samples

**Figure 1:**
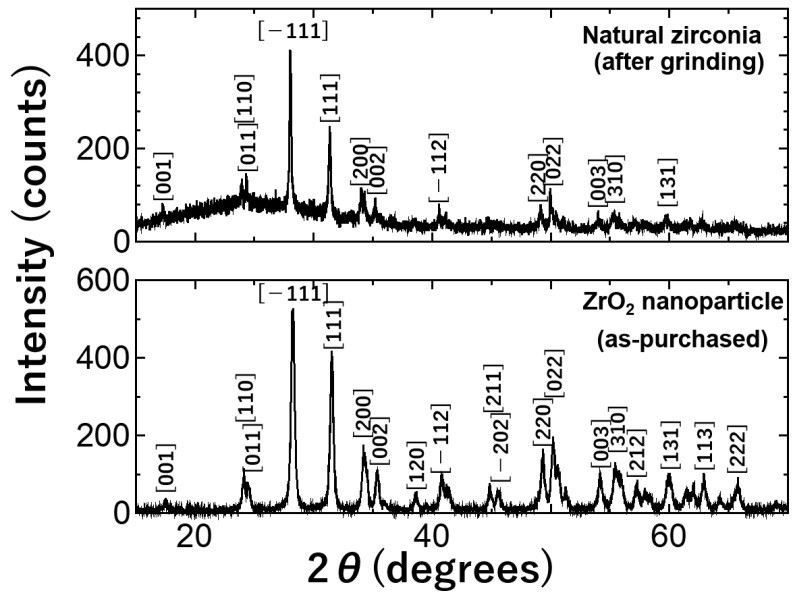
XRD patterns of natural zirconia samples measured after mechanical grinding and as-purchased synthetic zirconia nanoparticles. Miller indices are presented in the figure to demonstrate that most of the peaks correspond to the monoclinic phase.

In the present experiment, natural zirconia samples were ground using an agate mortar and pestle to prepare thin samples suitable for TEM observation. It is suspected that mechanical grinding may induce phase transformation from monoclinic to tetragonal [44]. Therefore, it is important to confirm the monoclinic phase of natural zirconia after mechanical grinding. As shown in Figure 1, the XRD peaks of the natural zirconia after grinding show that most of the peaks correspond to that of the monoclinic phase. The miller indices are presented based on previous XRD studies of monoclinic zirconia [45,46]. The XRD pattern of the as-purchased zirconia nanoparticles is also shown in the same figure, indicating that the peaks correspond to that of the monoclinic phase. Therefore, the samples are confirmed to be of the monoclinic phase. It is also confirmed that XRD peaks corresponding to the tetragonal phase are not recognizable in the XRD patterns.

### 3.2. Ion Tracks and Nanohillocks in Natural Zirconia

**Figure 2:**
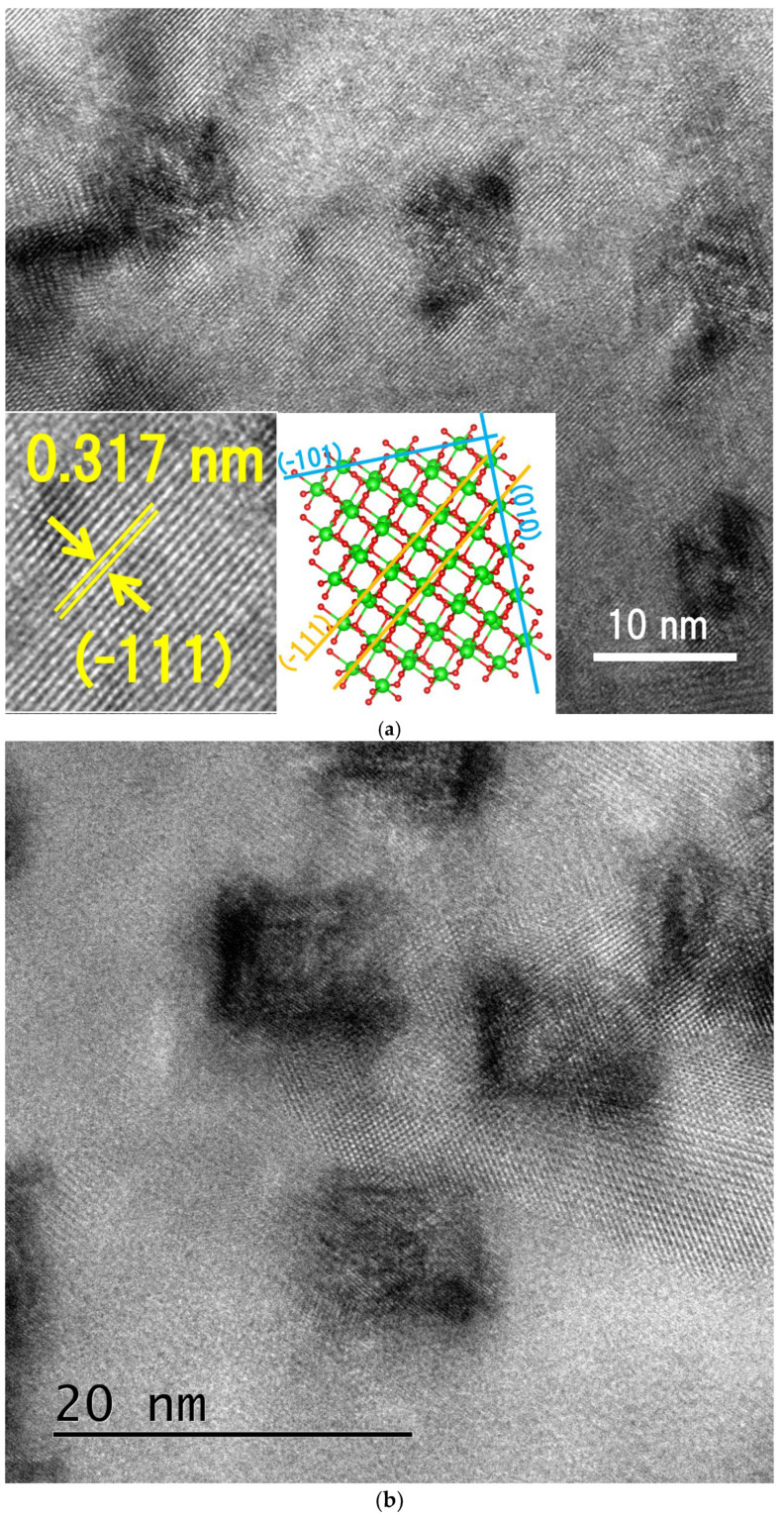

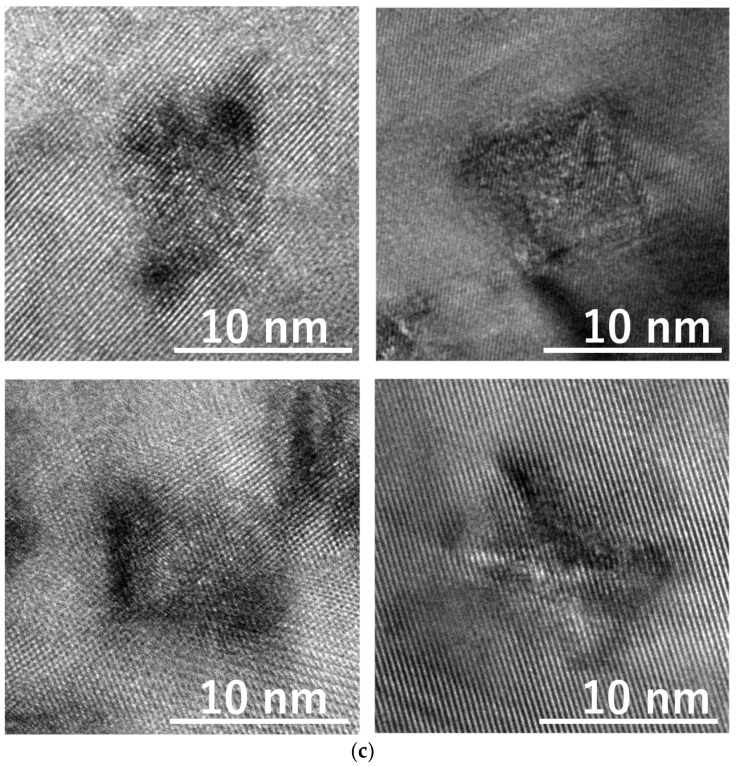
Bright-field images of ion tracks in natural zirconia irradiated with 340 MeV Au ions at normal incidence. Rectangular as well as parallelopiped strain contrasts are observed in (**a**). In the inset (**left**) of (**a**), magnified image of the matrix is shown, indicating the lattice spacing is 0.317 nm, which corresponds to that of (−111). In the inset (**right**) of (**a**), crystal structure of monoclinic zirconia is visualized using VESTA software Version 3.5.8, where green atoms indicate zirconium atoms and red atoms indicate oxygen atoms. It shows possible interpretation of crystal orientation of the TEM image. Rectangular strain contrasts are observed in another grain as shown in (**b**). Examples of ion track images in natural zirconia are shown in (**c**).

TEM images of the natural zirconia irradiated with a 340 MeV Au ion beam at a normal incidence are shown in Figure 2. While some of the ion tracks appear to have a parallelopiped shape (Figure 2a), rectangular cross-sections of ion tracks (Figure 2b) are imaged in most of the grains. All the ion tracks observed in the same grain have a similar shape and orientation. Most of the ion tracks exhibit a rectangular shape instead of circular shapes, indicating that their shape is determined by the relative angle between the crystal orientation and the irradiation direction. As shown in Figure 2c, the ion tracks have a dimension of approximately 10 nm. The ion tracks can be imaged as strained regions, and their lattice structure appears to be maintained. The lattice spacing and orientation of the strained regions (ion tracks) are similar to those of the matrix. As shown in the magnified TEM image of the matrix (left inset of Figure 2a), the lattice spacing can be estimated to be 0.317 nm, which corresponds to that of (−111) planes. Based on this observation, visualization of the crystallographic structure of the zirconia was attempted using the VESTA software Version 3.5.8 (right inset of Figure 2a). It is possible to interpret that the (−101) and (010) planes correspond to the sides of the rectangular track contrast. The possible interpretation of this analysis will be discussed later in the discussion section.

**Figure 3:**
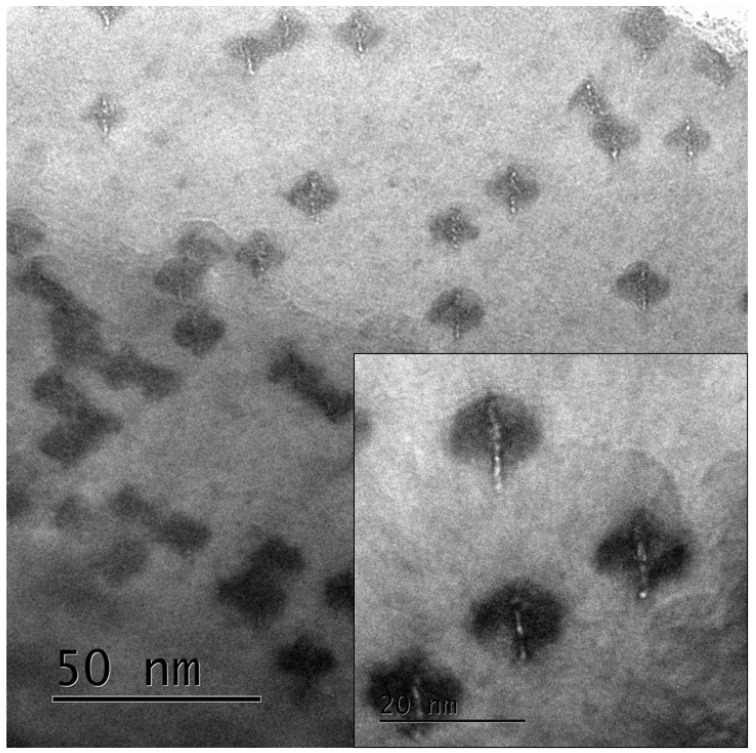
Bright-field image of ion tracks in natural zirconia irradiated with 340 MeV Au ions at normal incidence taken, where the sample is tilted in the microscope. The images were taken under under-focus conditions. The inset shows a magnified image of elongated ion tracks. The inset demonstrates that the length of elongated low-density cores is longer than that of the elongated strain fields.

When the sample is tilted in the microscope and observed under off-focus conditions, the strain contrast appears to be elongated, and line-like features can be imaged, as shown in Figure 3. The line-like contrasts suggest that the density of the core part of the ion tracks is lower than that of the matrix. The inset of the figure shows that the length of the low-density cores is longer than that of the elongated strain contrasts. The reason for this different elongation after the sample tilt will be discussed later.

It should be noted here the upper portion of the figure shows a clear contrast of the low-density cores that are imaged as Fresnel contrast [47], whereas they are not clearly imaged in the lower left portion of the figure. We believe that the atomic density of the low-density cores in the upper portion of the figure and that of those in the lower left corner of the figure are the same. However, it is likely that the volume fraction of the low-density cores to the undamaged matrix is different. The volume fraction of the low-density cores in the thin part of the sample is high, whereas that in the thick part of the sample is low. In the thick part of the sample, the low-density cores are surrounded by a relatively larger volume of undamaged matrix. Basically, Fresnel contrast is sensitive not only to the density difference, but also to the volume fraction between the (low-density) object and the matrix. Therefore, it is reasonable to interpret that the different features are closely related to the thickness of the sample.

**Figure 4:**
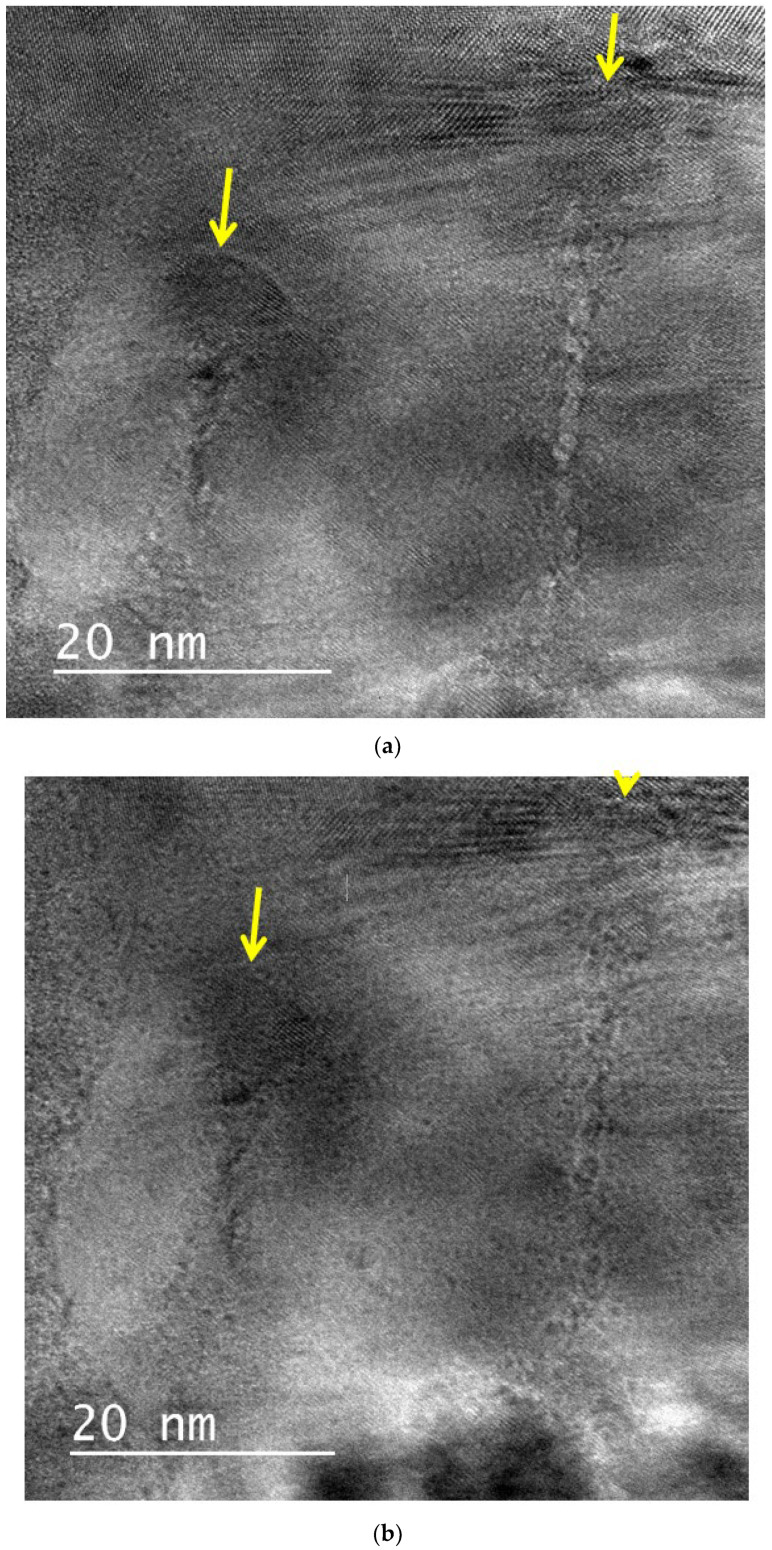
Bright-field images of ion tracks in natural zirconia irradiated with 340 MeV Au ions at inclined incidence. The irradiation direction is indicated by arrows. The images were taken under (**a**) underfocus and (**b**) overfocus conditions. Low-density cores in the vicinity of ion paths are visible as Fresnel contrasts.

Figure 4 shows images of a natural zirconia sample irradiated at a 45° inclined incidence relative to the normal incidence direction. Although the strained regions observed for the sample irradiated at a normal incidence are not clearly visible under off-focus conditions, the low-density cores aligned along the irradiation direction are visible. It appears that the low-density core comprises small fragments aligned along the ion paths. In the same image, semicircular contrasts located at the end of the line-like contrasts are also visible, indicating that nanohillocks are formed at the end of the ion tracks. The dimension of the nanohillocks is larger than that of the low-density core region.

**Figure 5:**
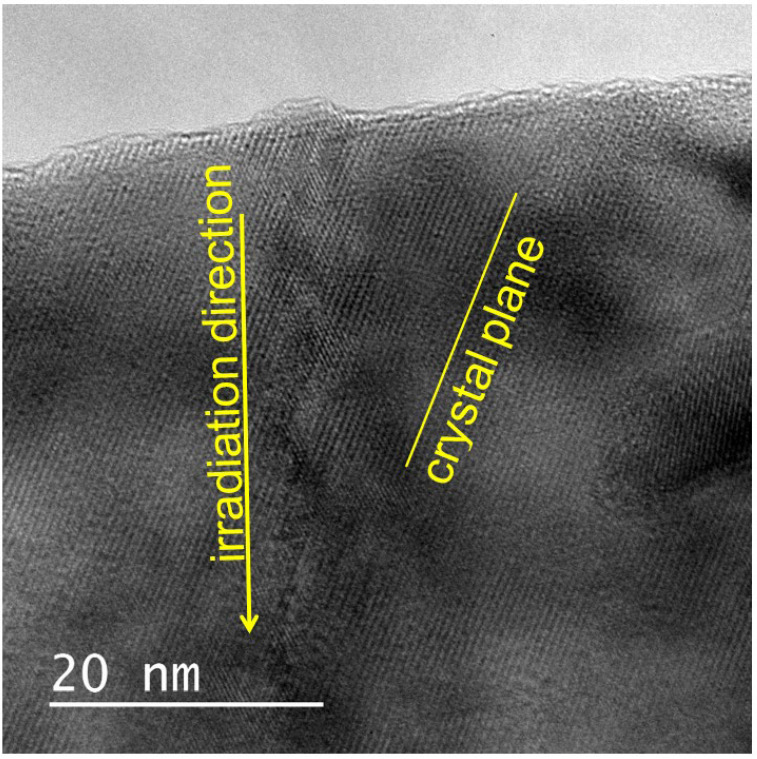
Bright-field images of ion tracks in natural zirconia irradiated with 340 MeV Au ions at inclined incidence. The irradiation direction is indicated by an arrow. A nanohillock is also imaged at the edge of the sample, whereas a zigzag-shaped strain field along the ion path is also recognizable.

Figure 5 shows that nanohillocks can be formed at the edge of the sample when irradiated at an inclined incidence. The diameter of the nanohillocks can be estimated to be approximately 10 nm. Although the strained region is recognizable in the vicinity of the ion path, the major parts of the strain contrast appear to be aligned along the crystal lattice plane. This shows that zigzag- shaped ion tracks can be formed, reflecting the lattice structure of the sample. Not only rectangular-shaped strain fields, but also these zigzag-shaped strain contrasts show that the irradiation-induced strain field is strongly affected by the crystal orientation of the lattice.

### 3.3. Ion Tracks and Nanohillocks in Zirconia Nanoparticles

**Figure 6:**
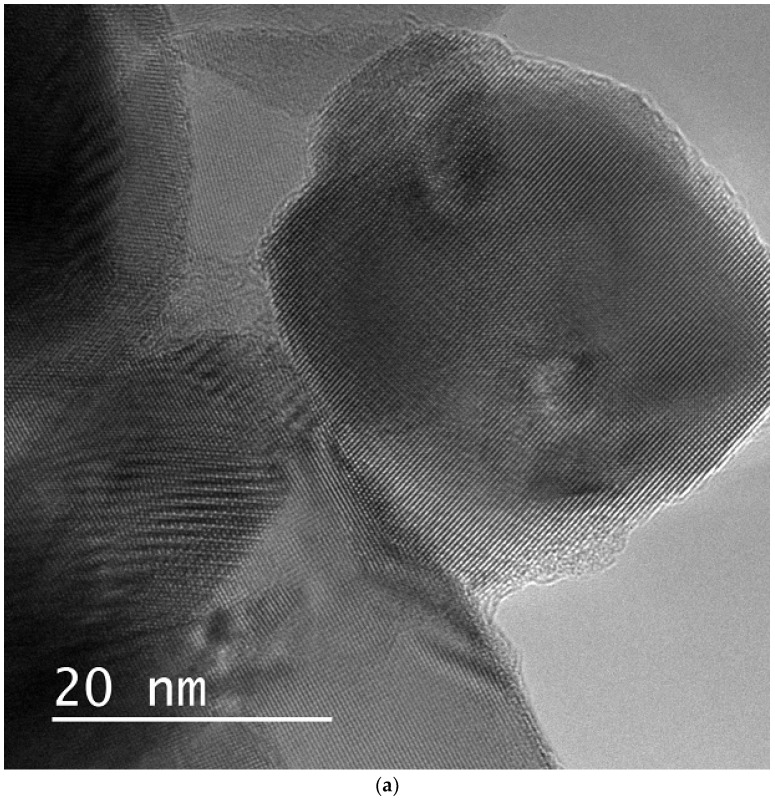

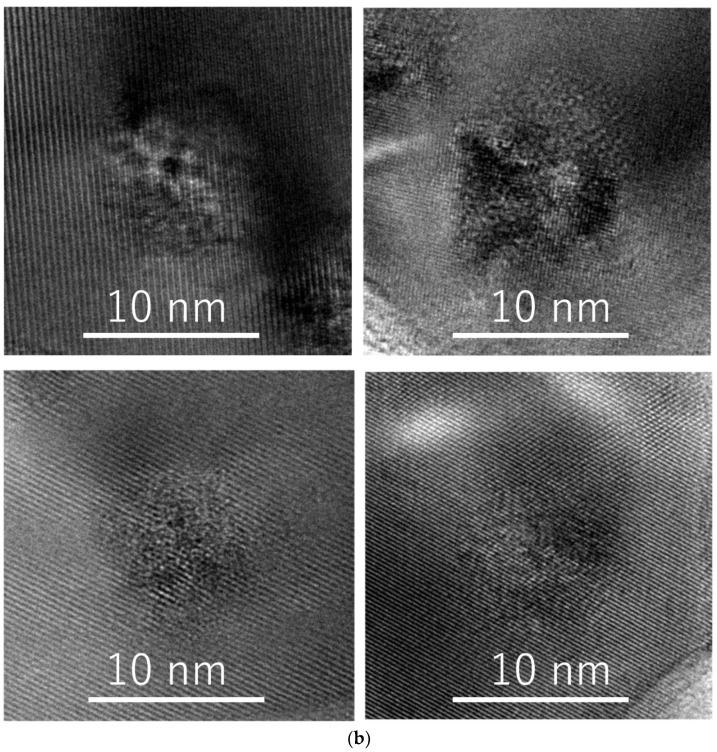
Bright-field images of ion tracks in zirconia nanoparticles irradiated with 340 MeV Au ions at normal incidence. Irregularly shaped strain contrasts (**a**) are observed. Examples of ion track images in zirconia nanoparticles are shown in (**b**).

For comparison, SHI irradiation-induced nanostructures in the zirconia nanoparticles were also observed. Figure 6a shows a TEM image of zirconia nanoparticles irradiated with a 340 MeV Au ion beam at a normal incidence. Ion tracks are imaged as strain fields and maintain their crystalline structure. Their size (~10 nm) is similar to that of the ion tracks in the natural zirconia, but they have different shapes. As shown in Figure 6b, the shape of the track cross-section is mostly irregular, although some of the ion tracks exhibit slight faceting reflecting the crystal lattice. The clear rectangular contrasts observed in the TEM samples of the natural zirconia are no longer observable in the zirconia nanoparticles. Moreover, the interface between the strained and matrix regions is not as clear as that in the natural zirconia. This suggests that the shape of the ion tracks is affected by the dimension of the sample or the distance between the ion track and the surface.

**Figure 7:**
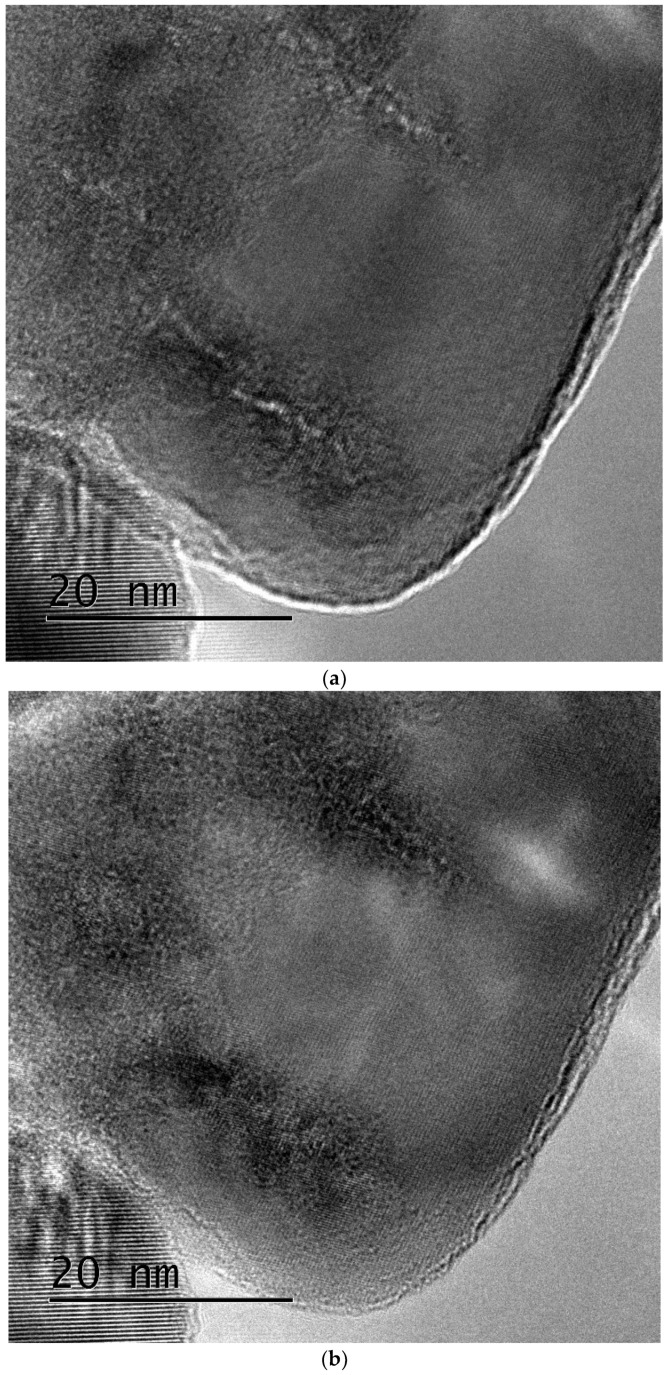
Bright-field images of ion tracks in zirconia nanoparticles irradiated with 340 MeV Au ions at normal incidence, where the sample is tilted in the microscope. The images were taken under (**a**) underfocus and (**b**) overfocus conditions. Low-density cores in the vicinity of ion paths are visible as Fresnel contrasts.

To confirm whether low-density core regions are also formed in zirconia nanoparticles, the sample was tilted slightly in the microscope. Consequently, low-density core regions similar to those observed in the natural zirconia are imaged. The core regions also appear as white (Figure 7a) and black (Figure 7b) line-like contrasts, depending on the focus condition.

**Figure 8:**
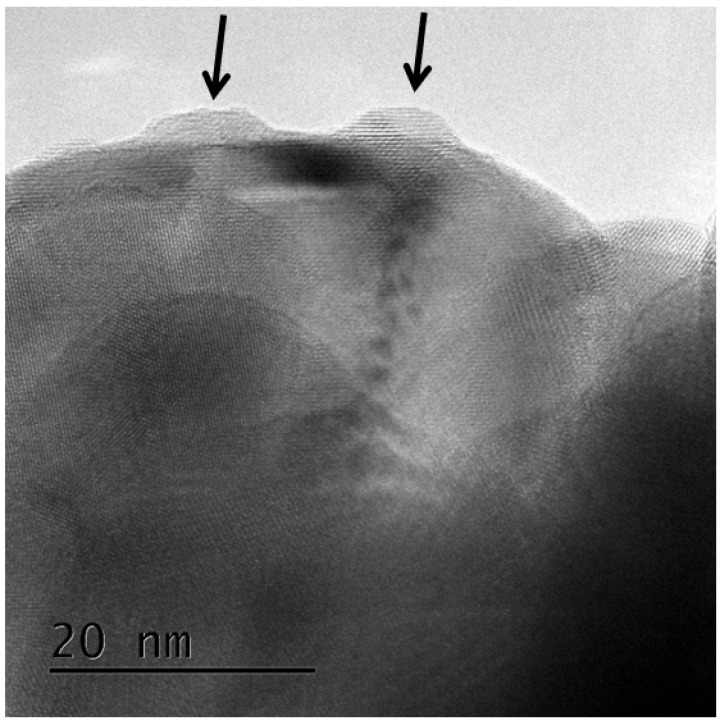
Bright-field images of ion tracks in zirconia nanoparticles irradiated with 340 MeV Au ions at inclined incidence. The image was taken under nearly on-focus conditions. The irradiation direction is indicated by an arrow. Nanohillocks are also imaged at the edge of the sample.

Figure 8 shows the TEM image of the zirconia nanoparticles irradiated at an inclined incidence (45° incidence relative to the normal incidence). Nanohillocks can be formed at the edge of the sample, enabling us to image their side view. The diameter of the nanohillocks is approximately 10 nm, and their size is similar to that of natural zirconia. The nanohillocks have a lattice structure similar to that of the matrix, suggesting that they are epitaxially recrystallized on the template of the matrix lattice. Ion tracks can be recognized as strained contrasts. Therefore, in both zirconia samples, the ion tracks are composed of two regions: the low-density core region and the surrounding strained region.

## 4. Discussion

### 4.1. Size of the Melt vs. Size of Nanohillocks

It is widely assumed that nanohillocks and ion tracks are formed because of transient local melting along the ion path [1,2,3]. On the basis of this assumption, it is important to calculate the dimension of the melt using the thermal spike model and compare it with that of the nanohillocks and ion tracks. The analytical thermal spike model (ATSM) proposed by Szenes is one of representative thermal spike models [48,49,50,51]. The ATSM mainly assumes that the ion-induced temperature increase ΔT(R, t) is approximated by a Gaussian distribution function. According to the model [48,49,50,51], the initial temperature increase as a function of the radial distance (R) from the ion trajectory at time = 0, ΔT(R, 0), is described by

(1) $$∆T\left(R,0\right)=\frac{gS_{e}}{\pi \rho ca^{2}\left(0\right)}\exp\left(-\frac{R^{2}}{a^{2}\left(0\right)}\right),$$

where ρ, c, g, and a(0) denote the density, specific heat, efficiency, and initial Gaussian width, respectively. The effective radius (R_e_) of the ion tracks can be calculated using the following conditions, assuming that the maximum melting region corresponds to the effective size of the ion tracks.

(2) $$∆T\left(R_{e},0\right)=T_{o}=T_{m}-T_{ir,}$$

where T_m_ and T_ir_ denote the melting and irradiation temperatures, respectively. Based on these assumptions, the following three equations are derived to describe the relationship between the track radius (R_e_) and electronic stopping power (S_e_):

(3) $$R_{e}^{2}=a^{2}\left(0\right)ln\frac{S_{e}}{S_{et}}\;\mathrm{for}\;S_{e}<2.7S_{et,}$$

(4) $$R_{e}^{2}=\frac{a^{2}\left(0\right)S_{e}}{2.7S_{et}}\;\mathrm{for}\;S_{e}>2.7S_{et}$$

where S_et_ denotes the threshold value of S_e_ for track formation, as expressed by Equation (5).

(5) $$S_{et}=\frac{{\pi \rho cT_{0}a}^{2}\left(0\right)}{g}.$$

S_e_ = S_et_ when the thermal energy of the spike (ε = gS_e_) is equal to the energy necessary to increase the maximum temperature to the melting point (T_m_). Many experimental data justify the validity of Equations (1)–(5) for track-forming insulators while assuming a(0) = 4.5 nm [48,49,50,51]. The efficiency g can be 0.4 or 0.17 for low or high ion velocities, respectively. Because 340 MeV Au ions have an energy of 1.7 MeV/u and are close to the low-ion-velocity regime, the efficiency g = 0.4 was used in this study. When the maximum molten size is calculated for zirconia, ρ = 5680 kg/m^3^, c = 0.47 kJ/Kkg, and T_m_ = 2988 K are used. On the basis of the above framework, the diameters of the maximum molten region are estimated to be D = 12.3 nm for zirconia irradiated with 340 MeV Au ion beams.

The present observation results show that the nanohillock diameter is in the order of 10 nm in both zirconia samples. It is similar to the diameter of the maximum molten region. The slightly smaller size of the nanohillocks may be due to the velocity effect. As demonstrated in ref. [50], g = 0.33 instead of 0.4 is possible when the energy is 1.7 MeV/u. These results support the hypothesis that nanohillocks are formed because of the surface protrusion of molten liquid created along the ion path in zirconia. These results are consistent with those of the previous studies on other ceramics [30], which showed that the nanohillock diameter is always similar to the diameter of the calculated molten region, irrespective of the amorphizability of the materials. The crystalline feature of the nanohillocks shows that the protruding part of the melt is epitaxially recrystallized, reflecting the crystal structure of the matrix as a template.

### 4.2. Anisotropic Recrystallization during Ion Track Formation

Because the ion tracks in both zirconia samples exhibit no amorphous features, the recrystallization process plays an important role in forming the crystalline ion tracks in zirconia. An anisotropic strain field is introduced, allowing us to visualize rectangular track contrasts. Because the shape and orientation of all the ion track contrasts in a sample grain are nearly the same, it is likely that the shape of the ion tracks is determined by the crystal orientation of the sample relative to the irradiation direction. This suggests that the molten region near the ion path is recrystallized anisotropically, reflecting the crystal orientation. A zigzag shape instead of a line shape of the ion tracks observed in the present observation also supports this anisotropic recrystallization.

According to the analysis using the VESTA software (right inset of Figure 2a), it is suggested that (−101) planes and (010) planes correspond to the sides of the rectangular contrast of the ion tracks. Both planes consist of alternate stacking of Zr layers and O layers. Since ZrO_2_ is predominantly ionic bonding [52], the character of bonding may play an important role in the recrystallization efficiency. It has been pointed out in previous studies [1,53] that materials with a higher degree of ionicity recrystallize easily. Since non-amorphizable ceramics include many ionic crystals, it is conceivable that long-range ionic forces rather than short-range covalent interactions contribute to rapid recrystallization [53,54]. Therefore, preferential recrystallization is possible along the direction perpendicular to the (−101) and (010) planes. However, at this moment, it is not clear why Al_2_O_3_ and MgO with a strong recrystallization efficiency exhibit irregularly shaped ion tracks, whereas in monoclinic zirconia, rectangular-shaped ion tracks are formed. Note that the rectangular shape of the track cross-section is unique to zirconia, because, in many other ceramics, a circular shape has been reported for track cross-sections [5,6,7,8,9,10,11,12,13,14,15,16,17,18,19,20,21]. In this regard, the recrystallization process of zirconia may somehow differ from that of other ceramics. Monoclinic zirconia exhibits anisotropic properties that reflect its anisotropic crystal structure. Many features of monoclinic zirconia, including elasticity, sound velocities, and minimum thermal conductivity, exhibit pronounced anisotropy [55]. According to ref. [56], the synthesis of monoclinic zirconia from amorphous zirconium hydroxide can lead to the formation of anisotropic rod-shaped nanoparticles, achieving a lower surface energy. The significant strain induced by the strong anisotropy of the thermal expansion of monoclinic zirconia [57] may also be related to the irradiation-induced anisotropic strain field.

### 4.3. Dimension of the Strain Field Contrasts

The rectangular shape of ion tracks has already been reported in a previous study on natural zirconia irradiated with 167 MeV Xe ions [32]. This result is consistent with the present observation of natural zirconia irradiated with 340 MeV Au ions. However, in the same study, the track dimension was reported to be 2.5 nm, whereas the present result shows that the size of the strain contrast is in the order of 10 nm. The size of the ion tracks observed in this study is significantly larger than that previously reported [32]. Therefore, it is necessary to reconcile these two seemingly contradictory results.

Note that the authors in ref. [32] reported a discrepancy between the size of rectangular contrasts (Figure 1b of ref. [32]) and that of tetragonal-phase regions (Figure 2 of ref. [32]). They stated that the former appears to be “considerably larger” than the latter. According to the literature, a possible interpretation for the larger rectangular contrast can be attributed to the inclined projection of the segments of smaller tetragonal-phase regions. This means that the segments of the tetragonal phase should be slightly offset from one another within the foil thickness (~40 nm) to image a large track contrast. Another interpretation presented in the literature is that the strong strain contrast surrounding the tetragonal phase allows us to image a larger strain contrast. Our findings support the latter interpretation. Our results show that (1) the dimension of the strained regions is as large as 10 nm and (2) the lattice structure of the strained track regions is similar to that of the matrix and is not significantly altered by track formation. Therefore, it is likely that the strain contrasts do not correspond to the tetragonal-phase region. Our results clearly show that strain fields as large as 10 nm are formed in natural zirconia.

### 4.4. Formation of Low-Density Cores

According to our results, the ion tracks are composed of two concentric regions: a low-density core region and a surrounding outer strained region. The formation of a low-density core region in the vicinity of the ion path has been reported previously in nonamorphizable materials such as NiO [58], GaN [59], and CeO_2_ [60]. In these materials, the recrystallization efficiency is high. According to these studies, the formation of low-density cores can be explained by material ejection toward the surface; thus, it is closely related to the formation of nanohillocks. If the recrystallization efficiency is high, the damage tends to recover rapidly during cooling after transient melting. However, once a material-deficient region is formed via material ejection, it is difficult to recover the damage during recrystallization, even if the recrystallization efficiency is high. Therefore, it is likely that low-density cores can be explained as residual nanostructures formed via material ejection and subsequent recrystallization.

Conversely, according to a previous molecular dynamics (MD) study on nonamorphizable materials (i.e., MgO and Al_2_O_3_ [19,20]), there is no signature of the formation of a low-density core along the ion path. This is probably because the MD simulations assume a bulk sample, and no significant material ejection toward the surface is assumed. According to another MD study on very thin nonamorphizable CaF_2_ [61], the track morphology is significantly affected by the film thickness due to the extrusion of molten matter through a formed liquid channel. Although the irradiation-induced nanostructure of CaF_2_ is not the same as that of zirconia, it is likely that the extrusion of molten matter plays an important role in forming the low-density regions of ion tracks.

There is a controversy about the interpretation of low-density objects created in the vicinity of ion paths. A paper on ion-irradiated NiO claimed that continuous nanopores are created in the vicinity of ion paths [58], whereas other papers on ion-irradiated CeO_2_ [16] and CaF_2_ [62] have claimed that only anion vacancies are introduced in the vicinity of ion paths. Similar contrasts are found in zirconia samples. It is difficult to determine via the present TEM observation whether the nanoobjects in the vicinity of the ion paths are voids/cavities or just clusters of point defects.

It is worth discussing the reason why low-density cores instead of tetragonal-phase regions are formed in this study. The different morphologies of the ion tracks are probably due to the sample conditions during irradiation. In previous studies [32,33], where tetragonal-phase cores were observed, TEM lamellae were prepared in the cross-section and plan view geometry after irradiation of the bulk sample. Conversely, in the present study, thin samples were irradiated, and the as-irradiated samples were observed using TEM. Therefore, it is possible that, in this study, the track formation process is significantly affected by material ejection, whereas, in the previous study, a tetragonal phase is formed in a deeper part of the sample, where material ejection from the surface is negligible. The study [32] stated that “it is typical for nonamorphizable materials such as ZrO_2_ to produce localized regions of lower density just below the surface from where the material was ejected to form the hillock. Presumably, this defected region inhibits the formation of the tetragonal phase”. Although the authors did not explicitly demonstrate the formation of low-density cores near the surface, they are aware of the difference between the morphology near and far from the surface. Therefore, material ejection is essential for the formation of low-density cores.

### 4.5. Particle Size Effect

The lateral dimension (the sample dimension perpendicular to the observation direction) of the natural zirconia sample tends to be larger than that of the zirconia nanoparticle sample. Therefore, it is expected that new insights related to the effect of particle size can be gained by comparing the track contrasts between the two samples.

**Figure 9:**
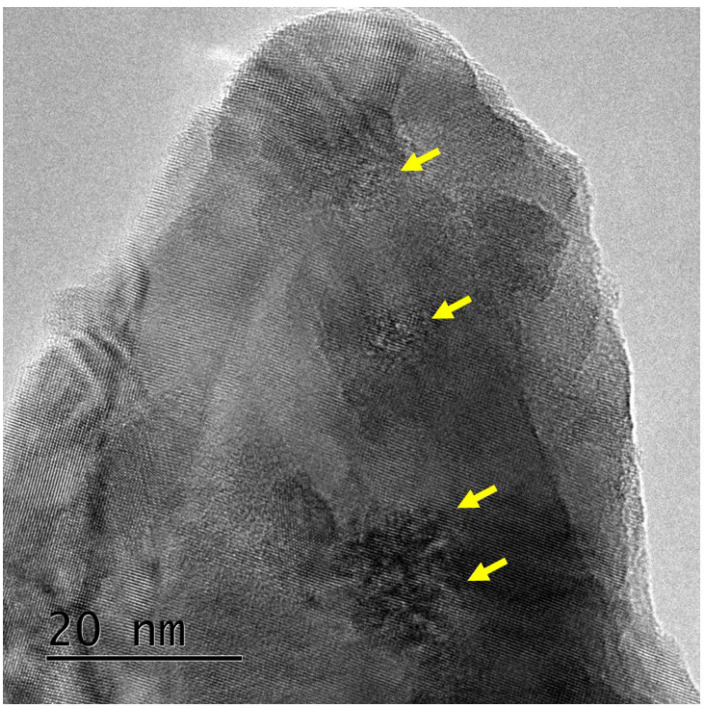
Bright-field image of ion tracks in a relatively small sample of natural zirconia irradiated with 340 MeV Au ions at normal incidence. The arrows are eye guides to show the position of the ion tracks. Irregularly shaped strain contrasts can be formed in a relatively small sample of natural zirconia.

A rectangular track cross-section reflecting the lattice structure was observed in the thin natural zirconia sample, whereas an irregularly shaped track cross-section was observed in the zirconia nanoparticle sample. Moreover, the interface between the ion tracks and the matrix is blurry and less clear in the zirconia nanoparticles. According to these results, the difference in the track cross-sections can be attributed to the effect of particle size. An irradiation-induced strain field can be easily relaxed in small samples such as zirconia nanoparticles because it is near the strain-free surface [63]. This interpretation is supported by the formation of irregularly shaped cross-sections of the ion tracks near the edge of the natural zirconia. Irregularly shaped ion tracks can be found near the edge of the TEM samples of the natural zirconia, as shown in Figure 9, although most of the ion tracks in the larger grains exhibit rectangular shapes in the natural zirconia. This indicates that irradiation-induced strain fields can be partially relaxed near the strain-free surface. Conversely, if the sample is large, the strained region is firmly constrained by the surrounding matrix, making it difficult to relax the irradiation-induced anisotropic strain field.

Another explanation for the absence of anisotropy is that the expulsion of molten matter is more significant in nanoparticle samples. In a recent study on nanoparticle Y_2_Ti_2_O_7_ [64], the isolated nanoparticles were more sensitive to SHI irradiation than larger samples, with an increasing susceptibility to track formation with a decreasing sample size. According to the literature, the higher sensitivity of nanoparticles can be explained by the presence of a surface upon which the expulsion of molten matter is significant. Here, the distance between the free surface and the molten matter is important. Molten matter tends to move toward the surface to relax the thermal stress applied due to the formation of molten matter. If the molten matter is near the surface, this tendency is naturally strong. Such a tendency can be also proven by so-called conical tracks [65,66], which demonstrates that the density of material deficiencies is high near the surface. According to the literature [66], the length of conical ion tracks is in the range of 70-80 nm. Since the distance between the surface and the molten matter is relatively short in nanoparticles, a higher density of material deficiencies can be introduced in nanoparticles.

Larger material deficiencies in zirconia nanoparticles can be also noticed as low-density pockets formed inside the ion tracks in some zirconia nanoparticles, as shown in Figure 6b. Crystalline tracks with low-density small pockets can be formed inside the ion tracks in nonamorphizable materials such as GaN [59] and TiO_2_ [65], where irregularly shaped ion tracks are formed. The formation of such low-density small pockets can inhibit complete recrystallization. Therefore, the absence of anisotropy in zirconia nanoparticles may be related to the higher density of irradiation-induced material deficiencies.

### 4.6. Absence of Anisotropic Strain Field Very Close to the Surface in Natural Zirconia

To examine the relative geometry of the low-density cores and anisotropic strain fields in the natural zirconia, the irradiated samples were tilted in the microscope, as shown in Figure 3. After tilting the sample, the low-density cores are imaged as elongated line-like contrasts, whereas rectangular strain contrasts also elongate in the same direction. As shown in the magnified image of the figure (inset of the figure), the line-like contrasts (i.e., low-density cores) are always longer than the elongated strain contrasts. This suggests that the low-density cores are likely formed from the front surface to the rear surface, whereas the latter is formed only in the middle part (deeper part) of the sample. The anisotropic strain field appears to be absent very close to the surface.

The reason for this absence of strain fields very close to the surface is currently unclear. Nevertheless, several explanations are possible. The formation of an anisotropic strain field is closely related to anisotropic recrystallization; therefore, the anisotropic recrystallization of the bulk sample is likely different from that near the surface. Strain fields may be easily relaxed near the surface, resulting in strain fields not being formed very close to the surface.

If the relative geometry between irradiation-induced strain fields and the free surface is a major factor affecting the formation of anisotropic strain fields, the absence of anisotropic strain fields in zirconia nanoparticles can also be due to the same reason. It is important to further study the effects of the surface and particle size. Although MD simulation has a certain limitation in simulating the irradiation-induced Frenkel defects of different charge states [67], MD simulations can be a help in clarifying the mechanism of anisotropic strain fields.

## 5. Conclusions

The TEM observation of natural monoclinic zirconia (baddeleyite) irradiated with 340 MeV Au ions shows the formation of nanohillocks with a diameter of approximately 10 nm. The similarity between the nanohillock diameter and the molten region diameter suggests that nanohillocks are formed because of the protrusion of the melt. The cross-sections of ion tracks are imaged as strained regions that maintain their crystalline structure. While some parallelopiped-shaped ion tracks are observed, the cross-section of most of the ion tracks is imaged as rectangular strain contrasts as large as 10 nm. These results strongly suggest that the molten region is recrystallized anisotropically, reflecting the crystal structure of the zirconia sample. It is also found that low-density track cores are formed in the center of the ion tracks. The formation of low-density track cores can be attributed to the ejection of molten matter toward the surface. A comparison between zirconia nanoparticles and larger natural zirconia samples shows that the interface between the strained track contrast and the matrix is less clear in the former than in the latter. These results suggest that the recrystallization process can be affected by the size of the irradiated samples.

## Data Availability

Data are contained within the article.
